# Editorial: Artificial Intelligence in Finance and Industry: volume II—highlights from the 7th European conference

**DOI:** 10.3389/frai.2023.1267377

**Published:** 2023-08-15

**Authors:** Andreas Henrici, Rudolf M. Füchslin, Peter Schwendner

**Affiliations:** ^1^School of Engineering, ZHAW Zurich University of Applied Sciences, Winterthur, Switzerland; ^2^European Centre for Living Technology (ECLT), Venice, Italy; ^3^School of Management and Law, ZHAW Zurich University of Applied Sciences, Winterthur, Switzerland

**Keywords:** Artificial Intelligence, deep learning, fintech, NMR, additive manufacturing, explainability

This Research Topic contains contributions from the seventh edition of the conference series on Artificial Intelligence in Finance and Industry. These conferences have been organized since 2016 at the Zurich University of Applied Sciences (ZHAW) in Switzerland and have attracted a large audience from industry and academia. Our conference series is motivated by the idea that the possibilities and challenges of AI applications in real-world contexts can be understood only by a bottom-up approach, i.e., by considering as many case studies as possible from as many different economic sectors as possible. As a university of applied sciences, we are convinced of the benefit and necessity of bi-directional communication between science and industry. Real-world problems often do not fit into the historically grown division of science into different sub-disciplines; our mission is to establish a true inter- and even transdisciplinary knowledge exchange. Moreover, given that almost all economic sectors now claim themselves to be technology-based, it is an urgent necessity to gain an overview of the specific challenges encountered in the practical implementation of abstract AI ideas. Proceedings of earlier conferences of the same series are contained in the Research Topic “*Artificial Industry in Finance and Industry: Highlights from 6 European COST Conferences*”; for an overview of its articles see Henrici and Osterrieder (2022). The present article collection contains two papers on AI applications in the industrial sector and two papers on AI applications in the financial sector, thereby highlighting the broad range of the topic in general and our conference specifically. The partial similarity of the underlying structures of these rather diverse applications emphasizes the usefulness and strength of these structures.

In the article “*Automatic Classification of Signal Regions in 1H Nuclear Magnetic Resonance Spectra*” by Fischetti et al., a supervised deep learning approach performs automated detection and classification of multiplets in one-dimensional NMR spectra. Even though the network was trained on artificial data, it also performs well on real-world data, which is an essential feature given the high costs of obtaining experimental data in this domain. Note that NMR spectroscopy is a spectroscopic technique that has been traditionally employed in various scientific fields for structure elucidation of chemical compounds and other substances. Even if automation of various steps of the NMR workflow has been attempted for a considerable time, deep learning techniques have only recently been introduced into NMR spectroscopy, with the article by Fischetti et al. being an important example of the potential of such techniques.

The article “*A Case Study for Unlocking the Potential of Deep Learning in Asset-Liability-Management*” by Krabichler and Teichmann applies deep learning techniques to a case study of a runoff portfolio of a stylized retail bank. The authors name their framework concept “Deep ALM” analogous to the “Deep hedging” approach of Bühler et al. (2019). They demonstrate the ability of their approach to outperform a static replication scheme.

The article “*Deep treasury management for banks*” by Englisch et al. refines the concept developed in the previous article and applies it to a specific case with actual data from a Swiss retail bank. The necessary yield curve scenarios are modeled in a HJM framework. The article demonstrates that such deep learning-based strategies can succeed in outperforming given benchmarks and complying with regulatory constraints. While acknowledging that the model presented in the article simplifies the real-world challenges of ALM, the flexibility of the model with regard to extensions is emphasized, as well as the need to keep explainability, especially when extending the ALM framework to more realistic scenarios.

The article “*Long-short term memory networks for modeling track geometry in laser metal deposition*” by Perani et al. presents an AI application in additive manufacturing. The issue to be modeled is over-deposition, a relevant phenomenon occurring in laser metal deposition and which should be controlled to enable automatisation of the additive manufacturing process. The authors investigate the generalization capabilities of LSTM networks in the sense that they show that a LSTM network which is trained on relatively simple track geometries shows good generalization capacities to more complex tracks, and even more so when random tracks are added to the training dataset.

One issue to be noted in these articles is the wide variety of application fields of AI methods, ranging from financial applications such as asset-liability management to industrial applications such as the analysis of NMR spectra and the control of over-deposition in additive manufacturing. Rather than being a sign of fragmentation, this seems to be a sign of the over-arching potential of these methods and the necessity of considering AI methods not as a separate research field but as a powerful toolbox whose usage has to be pondered by most industrial practitioners. Note also that neither of these studies is an isolated attempt to apply AI methods to problems in the analysis of NMR spectra or additive manufacturing, as can be seen by previous studies by the respective authors, see Perani et al. (2023) and Schmid et al. (2023), and other references in these papers.

Besides the mainly technical issues discussed in these Proceedings, and especially because of the many applications promising success prospects, the acceptance of AI methods among a wider audience also relies on their trustworthiness and the explainability of their results. Creating trust in the results of AI-based algorithms is an issue probably as important as increasing the accuracy and speed of the algorithms. Issues of human control over AI systems and shaping AI technology based on the need for explainability of AI-based decisions have already been discussed in articles of our previous conference proceedings, see Methnani et al. (2021) and Cerneviciene and Kabasinkas (2022), and will remain a crucial topic in the future.

Altogether, AI seems to be no longer mainly a Research Topic with some interesting applications, but a paradigm penetrating almost all data- and technology-based industrial sectors, whose success, however, not only depends on its technical power but also on the public trust in its results, which also shape eventual regulations such as the proposed EU AI Act.

## Author contributions

AH: Writing—original draft, Writing—review and editing. RF: Writing—review and editing. PS: Writing—review and editing.
